# Exposure to Tobacco-Specific Nitrosamines Among People Who Vape, Smoke, or do Neither: A Systematic Review and Meta-Analysis

**DOI:** 10.1093/ntr/ntad156

**Published:** 2023-08-24

**Authors:** Eve Taylor, Erikas Simonavičius, Ann McNeill, Leonie S Brose, Katherine East, Tim Marczylo, Debbie Robson

**Affiliations:** Addictions Department, Institute of Psychiatry, Psychology and Neuroscience (IoPPN), King’s College, London, UK; NIHR HPRU Environmental Exposures and Health, London, UK; Addictions Department, Institute of Psychiatry, Psychology and Neuroscience (IoPPN), King’s College, London, UK; Addictions Department, Institute of Psychiatry, Psychology and Neuroscience (IoPPN), King’s College, London, UK; NIHR HPRU Environmental Exposures and Health, London, UK; SPECTRUM Consortium, London, UK; NIHR ARC South London, Oxford, UK; Addictions Department, Institute of Psychiatry, Psychology and Neuroscience (IoPPN), King’s College, London, UK; SPECTRUM Consortium, London, UK; Addictions Department, Institute of Psychiatry, Psychology and Neuroscience (IoPPN), King’s College, London, UK; NIHR HPRU Environmental Exposures and Health, London, UK; Radiation, Chemical and Environmental Hazards, UK Health Security Agency (UKHSA); Addictions Department, Institute of Psychiatry, Psychology and Neuroscience (IoPPN), King’s College, London, UK; NIHR HPRU Environmental Exposures and Health, London, UK; SPECTRUM Consortium, London, UK; NIHR ARC South London, Oxford, UK

## Abstract

**Introduction:**

Smoking exposes people to high levels of Tobacco-Specific Nitrosamines (TSNAs), which include potent carcinogens. We systematically reviewed TSNA exposure between people smoking, vaping, and doing neither.

**Aims and Methods:**

Databases were searched between August 2017–March 2022, using vaping-related terms. Peer-reviewed articles reporting TSNA metabolites (NNAL, NNN, NAB, and NAT) levels in bio-samples among adults exclusively vaping, exclusively smoking, or doing neither were included. Where possible, meta-analyses were conducted.

**Results:**

Of 12 781 identified studies, 22 were included. TSNA levels fell substantially when people who smoke switched to vaping in longitudinal studies and were lower among people who vaped compared to smoked in cross-sectional studies. Levels of TSNAs were similar when comparing people who switched from smoking to vaping, to those who switched to no use of nicotine products, in longitudinal studies. Levels were higher among people who vaped compared to people who neither vaped nor smoked in cross-sectional studies.

When comparing people who vaped to smoked: pooled urinary NNAL was 79% lower across three randomized controlled trials and 96% lower across three cross-sectional studies; pooled NAB was 87% lower and NAT 94% lower in two cross-sectional studies. When comparing people who neither vaped nor smoked to people who vaped, pooled urinary NNAL was 80%, NAB 26%, and NAT 27% lower in two cross-sectional studies. Other longitudinal data, and NNN levels could not be pooled.

**Conclusions:**

Exposure to all TSNAs was lower among people who vaped compared to people who smoked. Levels were higher among people who vaped compared to people who neither vaped nor smoked.

**Implications:**

As well as TSNAs, there are many other toxicant exposures from smoking and vaping that can increase the risk of disease. However, it is likely that the reduced exposure to TSNAs from vaping relative to smoking reduces the risk to health of those who use vaping products to quit smoking. Future high-quality research, with robust definitions of exclusive vaping and smoking, and accounting for TSNAs half-lives, is needed to fully assess exposure to TSNAs among people who vape.

## Introduction

In 2019, approximately 7.7 million deaths were attributable to tobacco smoking worldwide, 65 000 in England.^1,2^ Most of these deaths were from smoking-related cancers, cardiovascular, and respiratory diseases. Smoking has been linked to approximately 15 different cancers, with 72% of lung cancers and 15% of all cancer cases in the United Kingdom estimated to be attributable to smoking.^3^

Tobacco-specific nitrosamines (TSNAs) are a group of toxicants, which include the main carcinogens in tobacco and tobacco smoke.^4^ Unlike other carcinogens found in cigarettes, for example, heavy metals, TSNAs are thought to be specific to tobacco. They are formed through nitrosation of nicotine alkaloids during the tobacco curing and fermentation process.^5^ The main TSNAs are: 4-(methylnitrosamino)-1-(3-pyridyl)-1-butanone (NNK) and its metabolite 4- (Methylnitrosamino)-1-(3-pyridyl)-1- butanol (NNAL) formed through nitrosation of nicotine; Nʹ-nitrosonornicotine (NNN) formed through nitrosation of nornicotine; Nʹ-nitrosoanabasine (NAB) formed through nitrosation of anabasine; and Nʹ-nitrosoanatabine (NAT) formed through nitrosation of anatabine Supplementary Table S1). Both NNK and NNN are classified as group 1 carcinogens by the International Agency for Research on Cancer, meaning there is sufficient evidence to classify them as carcinogenic to humans. NAB and NAT are “not classifiable as to its carcinogenicity to humans,” meaning that their carcinogenic potential is unknown and there are significant gaps in research.^4^ As with other procarcinogens, TSNAs go through a process of metabolic activation to react with DNA and form DNA adducts (a covalent binding product of a carcinogen or related substance or its metabolite to DNA), which can lead to mutations. In addition, long-term smoking or persistent exposure to secondhand tobacco smoke can interrupt DNA repair, preventing the removal of DNA adducts. Together these processes increase the likelihood of DNA damage, genetic mutations, and the development of cancers.^6^ Specifically, NNK and NNN have been associated with lung, liver, esophageal, and pancreatic cancers in animal studies.^7^ NNK has also been reported to have a dose-dependent effect on the risk of lung cancer in humans.^8^

TSNAs are present in all forms of smokeless and combustible tobacco, although their levels can vary between brands and manufacturing process methods.^9^ Amongst people who smoke, the levels of TSNAs in urine are dose-dependent, such that levels increase with a greater number of cigarettes smoked.^4,10^ Levels of NNAL, which can also be metabolized to form DNA adducts, have been found to drop substantially when people stop smoking.^11^ However, because of the long half-life (10–45 days),^11,12^ and idiosyncratic metabolism, NNAL has a wide variation in total body clearance, with one study reporting detectable levels of urinary NNAL over eight months after participants reportedly quit smoking.^12^ NNAL levels can also be influenced by secondhand exposure, for example as a consequence of living with someone who smokes.^13^

Nicotine vaping products (also called e-cigarettes) do not contain tobacco, help people quit smoking, and likely reduce exposure to carcinogens among people who smoke who completely switch to vaping.^14,15^ TSNA levels may be very low in e-liquid because of the purified tobacco-derived, or synthetic, pharmaceutical-grade nicotine that is typically used.^5^ In a study involving e-liquids that were fortified with nitrates and minor alkaloids (to mimic exposures from e-liquids containing impurities), NNK and NNN were detected when liquids were heated to temperatures above 150°C.^16^ Therefore, TSNA exposure may occur from vaping if there are impurities in the nicotine that is used. A systematic review of vaping products and aerosol toxicants found that the levels of all TSNAs in e-liquids and the aerosol (or vapor) emitted from vaping devices were near or below the limit of detection,^17^ and are substantially lower than levels found in cigarettes.^18^

Measures of toxicant levels in e-liquids or vapor are not always accurate predictors of human exposure levels that are accumulated during repeated vaping or secondhand exposure, as they do not take into account product-level (eg, device) or individual-level variables (eg, frequency or intensity of vaping, or individual metabolism).^19^ The U.S. National Academies of Science Engineering and Medicine (NASEM report) in 2018 concluded that most vaping products contain and emit numerous potentially harmful substances, but toxicant exposure from vaping was significantly lower than from combustible tobacco cigarettes.^5^ Other systematic reviews also reported reductions in NNAL levels among people who smoke who completely switch to vaping.^20–22^ A comprehensive systematic review commissioned by the Office for Health Improvement and Disparities (OHID) in England, examined studies from August 1, 2017 (the end date used in the search for the NASEM report) to July 1, 2021 and concluded in 2022 that levels of TSNA were substantially lower among people who vape compared to people who smoke.^23^ Since the cutoff date of the OHID-commissioned review (July 2021), further studies providing longer-term data have been published. It is important to regularly review the evidence regarding TSNA exposure from vaping, due to the rapidly evolving product market and the wider variety of products now available. Newer vaping products can increase the amount of aerosol generated and the bioavailability of nicotine.^20^ Therefore, we aimed to update the evidence by systematically reviewing and meta-analyzing levels of TSNAs among people who exclusively vaped compared to people who exclusively smoked and those who were not currently vaping or smoking.

## Method

This review updates evidence presented in a larger report on health risks and effects of vaping.^23^ The protocol for the original review was registered on the International Prospective Register of Systematic Reviews, PROSPERO (CRD42020215915).

## Eligibility Criteria

Randomized controlled trials (RCTs), non-randomized intervention longitudinal studies (where participants can choose what group they are assigned to, or all participants are assigned to the same group and participants are followed up over time), observational longitudinal studies (where there is no intervention and participants are followed up over time), cross-over studies (where all participants participate in all study conditions in succession), single acute exposure studies and cross-sectional studies, were included to present a full picture of the evidence. Qualitative studies were excluded. Other literature (e.g., research posters, conference abstracts, PhD theses, research letters) were also excluded as these are not peer-reviewed in the same way journal articles are.

*Participants*: Adults aged ≥18 years.

*Intervention*: Participants who exclusively vaped at baseline and/or follow-up. For RCTs and non-randomized longitudinal studies, where participants who were allocated to a vaping arm but were still smoking at follow-up, or where secondary analyses had not been conducted among participants who were exclusively vaping, were excluded.

Due to different smoking and vaping frequencies among people who concurrently smoked and vaped (“dual users”^24^), data on TSNA levels among dual users were not included.

*Comparator:* Participants who exclusively smoked tobacco cigarettes or were not using a nicotine or tobacco product (“non-users”) at baseline and/or follow-up.

*Outcome*: Levels of biomarkers of TSNA exposures and their metabolites (eg, NNK, NNAL, NAB, NAT, and NNN) in bio-samples of urine, blood, saliva, or hair.

Studies were grouped by study design and bio-sample (urine, saliva), and comparisons (people who vape vs. smoke, people who vape vs. neither vape nor smoke).

Follow-up in longitudinal studies was grouped as: short-term (less than 8 days); medium-term (8 days to 12 months); long-term (more than 12 months).

### Search Strategy

We conducted a systematic review of literature identified in five electronic databases (PubMed, Embase, PsycInfo, CINAHL, and Medline). Databases were searched using e-cigarette-related terms (Supplementary Table S2) from August 1, 2017 to July 1, 2021. The search was then updated to include studies published between July 2, 2021 and March 18, 2022 using the same methods. Search terms were based on those previously used by McNeill et al.^25,26^

### Screening and Extraction

Titles, abstracts, and full-text papers were screened by two of the three reviewers (ET, ES, KE). For RCTs and non-randomized longitudinal studies, only data from per-protocol analysis was extracted. Any discrepancies in the selected studies were discussed between the reviewers with support from a third reviewer. Data were extracted independently by one of two reviewers (ET, ES) with a subsample checked for accuracy by a second reviewer (DR or AM).

### Risk of Bias Assessment

Risk of bias was assessed by one reviewer (ET, ES, DR, or AM), with 20% assessed by two reviewers. The following risk of bias tools were used: The Cochrane Risk of Bias tool (RoB2) for RCTs,^27^ the ROBINS-I tool for longitudinal studies with an intervention,^27^ the Newcastle-Ottawa tool^28^ for observational longitudinal studies, and the BIOCROSS tool for cross-sectional studies^29^ (Supplementary Tables S3–6).

### Synthesis Methods

TSNA biomarker levels, as well as results of statistical significance testing for comparisons between people who vaped, smoked, and did neither were extracted and tabulated (Supplementary Tables S7–11). For each study, the percentage difference was calculated between people who either vaped, smoked, or did neither. For cross-sectional comparisons, levels among people who vaped were reported as a percentage difference of levels among people who smoked (1−(Vaping level/ Smoking level)) × 100, and levels among non-users were reported as a percentage difference of levels among people who vaped (1−(Non-use level/ Vaping level)) × 100. For within-group longitudinal comparisons, follow-up levels were reported as percentage of baseline levels (1−(Follow-up/ Baseline)) × 100.

### Meta-Analysis

Because of methodological heterogeneity, we developed criteria to identify studies suitable for meta-analyses (see also McNeill et al., 2022).^23^ Reasons for inclusion and exclusion of each study are outlined in Supplementary Table S12. These criteria included:

- People who vaped, or smoked, had been vaping, or smoking, at least weekly (as less frequent vaping might underestimate exposure to most toxicants that have shorter half-life characteristics).
- Data were available as means and standard deviations or confidence intervals. Studies that reported modes and interquartile ranges or least squares means could not be pooled.
- Use of similar biomarker analysis techniques. For example, gas chromatography methods could not be pooled with ELISA kit because of differences in sensitivity.
- Urinary data had been adjusted for concentration eg, for creatinine or excretion over 24 hours.
- If two or more studies reported on the same data source for the same time period, for example, data from the same survey wave, only the study with the largest sample size was included.

Biomarker levels reported on both the arithmetic scale and geometric scale were converted to their natural log.^30^ Generic inverse-variance method using random effects models was used to pool log means and log standard deviations. Studies were weighted depending on sample size and standard deviations.^31^

To better communicate the log-transformed between-group mean differences (LMD) in meta-analyses, the geometric mean ratios were calculated which allowed us to evaluate the biomarker level differences between groups. Geometric mean ratios were calculated by exponentiating the log mean differences and then converting them to a percentage difference to aid interpretation. We assessed statistical heterogeneity between studies using the *I*^2^ statistic. Analyses were conducted using RevMan 5.4.1 software.^32^

### Key Differences From the Preregistration and OHID-Commissioned Review

**Figure 1.**
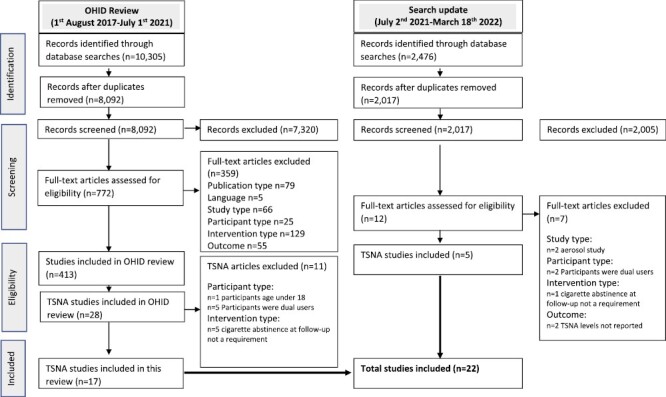
Preferred reporting items for systematic reviews and meta-analyse flowchart.

There are some methodological differences between the OHID-commissioned preregistration,^33^ report,^23^ and this review. As this review only discusses TSNAs, levels of which are highly sensitive to tobacco exposure, methodologies were tailored as follows to provide more robust analysis of relative and absolute exposure. In contrast to the preregistration, studies that included participants who were under the age of 18, secondhand exposure as the intervention, or participants who dual used, or used any other nicotine or tobacco product as the comparator were excluded. In contrast to McNeill et al.,^23^ this review excluded studies where it was not a requirement for people who vaped to be abstinent from smoking (Figure 1, excluded due to intervention). Biomarker levels in people who do not vape or smoke are presented here as percentage of levels among people who vape. This differs from the OHID-commissioned report where levels among people who vape were presented as percentage of levels among people who do not vape or smoke. This was to aid interpretation, by ensuring relative and absolute levels are presented on the same scale. Also, meta-analyses of longitudinal studies only included levels from follow-up waves, and where stated, levels were used from people who vaped or smoked daily for cross-sectional meta-analyses for consistency across comparison groups. If levels for daily use were not provided, levels from at least weekly use were analyzed.

## Results

The search identified 12 781 studies, of which 22 were included in this review, including five additional longitudinal studies from the updated search (See Figure 1- PRISMA flowchart). All included studies reported urinary TSNA levels, with one also reporting on salivary levels.^34^ Longitudinal studies included eight RCTs^35–42^ two non-randomized intervention studies^43,44^ and two observational studies.^45,46^ Ten cross-sectional studies^34,47–55^ were also included. No cross-over studies were identified. Funding sources for each study are available in Table 1 and Supplementary Table S13.

**Table 1.** Longitudinal Study Characteristics and Outcomes

Author year	Total *N*	Time	CO for inclusion	CPD at baseline	% Change from baseline					Risk of bias
					Follow-up group *N*	NNAL	NNN	NAB	NAT<	
RCT										RoB2
Jay 2020^40^^*^	90	5 days	CO ≥12 ppm	CPD *M* = 16.2 SD = 3.6	Vaping *n* = 60 Nonuse *n* = 11	↓68% ↓67%	↓61% ↓99%			Some concerns
Round 2019^39^^*^	158	5 days		CPD *M *= 14.4	Vaping^+^ *n* = 38 Vaping^+^ *n* = 40	↓59% ↓55%	↓87% ↓92%			Some concerns
Cohen 2021^37^^*^	295	6 days	CO >10 ppm	CPD *M* = 18 SD = 5.2	Vaping *n* = 188 Nonuse *n* = 23	↓66% ↓64%	↓93% ↓97%	↓89% ↓87%	↓99% ↓98%	Some concerns
McEwan 2021^42^^*^	148	7 days	CO >10 ppm	CPD *M* = 20.1 SD = 4.83	Vaping *n* = 28 Nonuse *n* = 29	↓65% ↓68%	↓77% ↓80%			Some concerns
Morris 2022^36^^*^	80	14 days	CO >10ppm	CPD > 10	Vaping^+^ *n *= 14 Vaping^+^ *n* = 11	↓73% ↓79%	↓92% ↓89%			Some concerns
Pulvers 2020^41^	186	6 weeks	CO >5ppm	CPD *M* = 12.1 SD = 7.2	Vaping *n* = 32	↓95%				Some concerns
Hatsukami 2020^38^	264	8 weeks	CO ≥8 ppm	CPD > 5	Vaping *n* = 57	↓53%				Some concerns
Edmiston 2022^35^^*^	150	24 weeks		CPD *M* = 17.6 SD = 5.0	Vaping^+^ *n* = 48 Vaping^+^ *n* = 50	↓84% ↓73%				Some concerns
Non-randomized intervention study										ROBINS-I
Goniewicz 2017^43^	20	2 weeks		CPD *M* = 16 SD = 9	Vaping *n* = 9	↓72%				Moderate
Pulvers 2018^44^	40	4 weeks		CPD *M* = 8.8 SD = 6.53	Vaping n = 6	↓97%				Moderate
Observational longitudinal										Newcastle-Ottawa
Anic 2022^46^	2475	12 months		CPD *M* = 11 CPD *M* = 5	Vaping *n* = 28 Nonuse *n* = 188	↓93% ↓85%	↓83% ↓44%	↓89% ↓65%	↓94% ↓75%	7/9
Dai 2022^45^	3211	12 months		CPD *M* = 16.7	Vaping *n* = 32 Nonuse *n* = 246	↓92% ↓84%	↓82% ↓44%	↓90% ↓63%	↓96% ↓74%	7/9

+ Results were separated by vape flavor, see Supplementary Table S6 for details.

^*^Denotes studies funded by the tobacco industry.

CPD = cigarettes per day, CO = carbon monoxide, M = mean, SD = standardized deviation, ↓ indicates % decrease between baseline and follow-up.

Unless otherwise stated, results indicate urinary levels.

Across the 12 longitudinal studies, ages ranged from an average of 30 years^44^ to 47 years,^38^ with between 27%^44^ and 60%^43^ females. All eight RCTs, and one non-randomized longitudinal study, required participants to be smoking daily, which was bio-verified using breath carbon monoxide in 6 studies.^36–38,40–42^All participants in longitudinal studies vaped or smoked daily or non-daily ad libitum. Study characteristics are outlined in Supplementary Table S7.

Across the 10 cross-sectional studies, ages ranged from an average of 31 years^34^ to 50 years,^54^ with 19%^55^ to 73% female participants.^34^ There was some variation in the measurements of participants’ frequency of vaping, smoking, and nonuse. Three studies of bio-verified use used breath carbon monoxide^47,54,55^(Table 1 and Supplementary Table S10).

### Risk of Bias in Included Studies

Of the eight RCTs, all had some concerns (Supplementary Table S3). Of the two non-randomized longitudinal studies, both were considered to have moderate risk of bias (Supplementary Table S4). Of the two observational longitudinal studies, both were considered as good quality (Supplementary Table S5). Of the 10 cross-sectional studies, most were considered good quality, with scores between 10^54^ and 16^55^ out of a maximum score of 20 on the BIOCROSS tool (Supplementary Table S6).

Unless otherwise stated, all studies included in the results discuss urinary findings.

## Smoking Versus Vaping

### NNAL Exposure

#### Within-Group Changes

In eight RCTs where people who smoked at baseline switched to vaping, NNAL was reduced significantly, in four short-term studies^37,39,40,42^ by 55% to 68%, and in four medium-term studies by 53% to 95%^35,36,38,41^(Table 1). Short-term use studies, which ranged from five to seven days, were all conducted in research facility where access to tobacco cigarettes or e-cigarettes were controlled by the research team. One 14-day medium-term study, Morris et al.,^36^ was also conducted in a research facility. The three other medium-term studies ranged from 6 to 24 weeks and all utilized carbon monoxide monitoring to ensure people who were randomized to vaping were not also smoking^35,38,41^(Table 1 and Supplementary Table S7).

Two non-randomized longitudinal studies^41,43^ reported a significant reduction in NNAL levels, one by 72% at 2 weeks^43^ and the other by 97% at 4 weeks^41^ (Supplementary Table S7). Two observational longitudinal studies investigated levels of NNAL among people who smoked and people who vaped using wave 1 (baseline) and wave 2 (12 months) of the PATH survey.^45,46^ NNAL levels among those who smoked and had switched to vaping fell significantly, by 92% and 93% (Table 1 and Supplementary Table S7).

#### Between-Group Differences

Compared to people who continued to smoke at follow-up, NNAL levels among people who switched from smoking to vaping at follow-up were 74% lower after 5 days,^40^ and 72% lower after 7 days of vaping,^42^ in studies conducted in research facilities. After medium-term use, levels were 93% lower after 6 weeks,^41^ 28% lower after 8 weeks,^38^ and 71%–85% lower after 24 weeks of vaping^35^ (Supplementary Table S7). Two studies did not report sufficient data on levels for those who smoked to provide comparisons.^36,37^ Among studies that tested for significance,^35,41,42^ all reported that differences were significant between people who vaped compared to those who smoked.

#### Meta-Analyses Between-Group Differences

**Figure 2.**
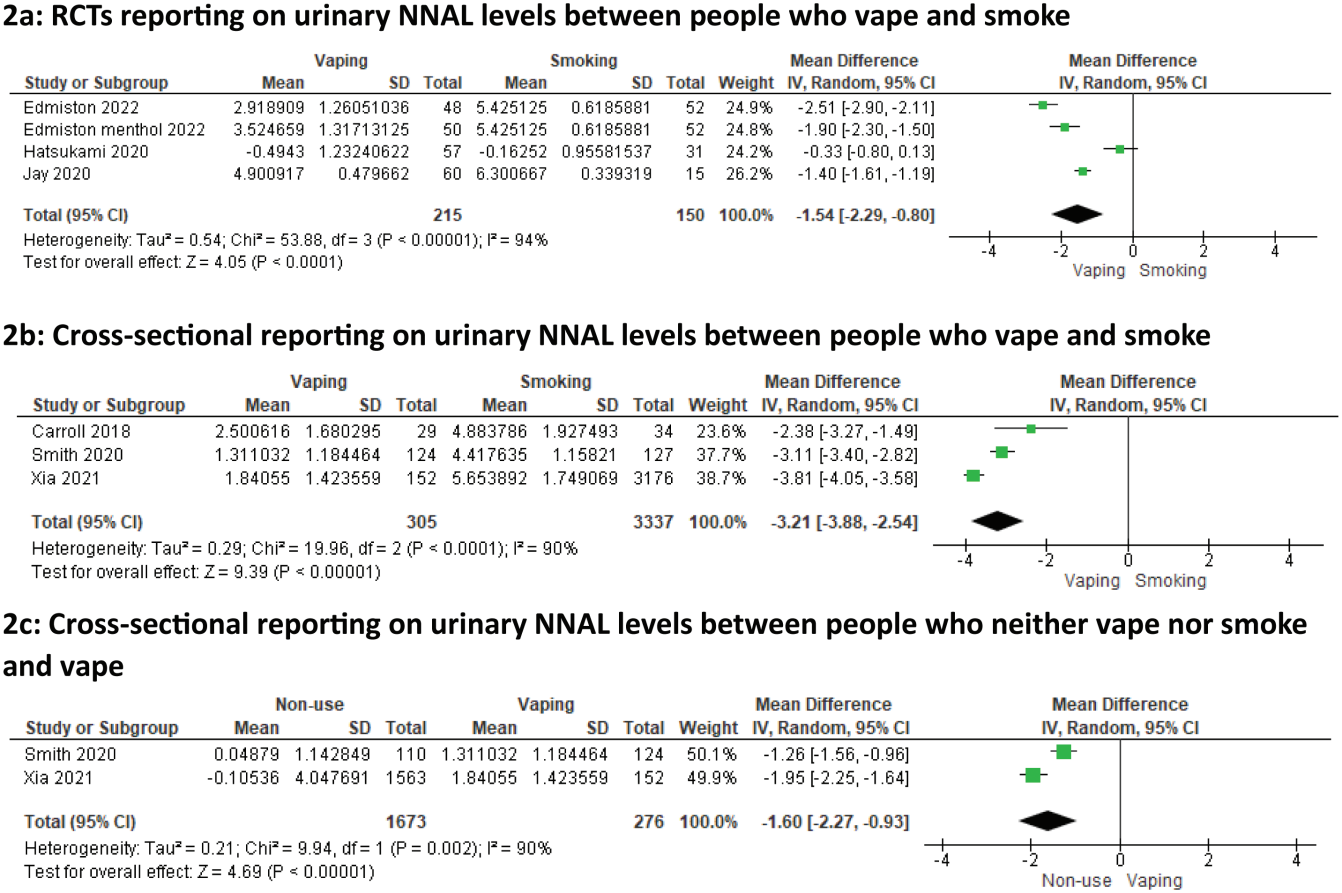
Meta-analysis of studies reporting on urinary NNAL levels between people who vape, smoke, and do neither. (A) Randomized controlled trials reporting on urinary NNAL levels between people who vape and smoke. (B) Cross-sectional reporting on urinary NNAL levels between people who vape and smoke. (C) Cross-sectional reporting on urinary NNAL levels between people who neither vape nor smoke and vape.

Of the eight RCTs comparing NNAL levels in vaping and smoking groups, two did not provide a smoking comparison group at follow-up, and three did not provide data in a form that could be pooled (Supplementary Table S12). Meta-analyses were undertaken with data from the remaining three studies comparing vaping and smoking groups after 5 days,^40^ 8 weeks,^38^ and 24 weeks of use^35^(Figure 2). The pooled geometric mean NNAL levels were 79% lower among people who vaped than among people who smoked (LMD = −1.54, 95% CI: −2.29, −0.80, *p* < .001). Heterogeneity was high at *I*^2^ = 94%, but, as levels were lower among those who vaped compared to those who smoked across the three trials, the direction of the difference was consistent.

#### Cross-Sectional Studies

Nine cross-sectional studies compared NNAL levels between people who vaped or smoked.^34,47,48,50–55^ Eight studies found that NNAL levels were significantly lower among people who vaped compared to smoked, by between 52%^52^ and 98%.^50^ Coleman et al., reported levels to be 93% lower among people who were non-pregnant and vaped, and 92% lower among pregnant people who vaped compared to smoked; however, neither comparison was tested for significance.^48^ Oliveri and colleagues reported marginally higher NNAL levels among people using cartridge vaping devices compared to those using tank vaping devices; however, this was not tested for significance^52^(Table 2 and Supplementary Table S10).

**Table 2.** Cross-Sectional Study Characteristics and Outcomes

Cross-sectional	*N*	Frequency of use	Difference between vaping and smoking				Difference between nonuse and vaping				Risk of Bias *(Biocross)*
			NNAL	NNN	NAB	NAT	NNAL	NNN	NAB	NAT	
Bustamante 2018^34^	Smoking *n* = 20 Vaping *n* = 20 Nonuse *n* = 19	Daily Daily <100 cigs in lifetime	95%↓ 85%↓ (saliva)	99%↓			99%↓ 98%↓(saliva)	0%98%↓ (saliva)			15/20
Carroll 2018^47^	Smoking *n* = 27 Vaping *n* = 23	Daily and CO > 6 Daily and CO =<6ppm	91%↓								13/20
Coleman 2021^48^	Smoking *n* = 84 Vaping *n* = 27	Some days/every day Some days/every day	Pregnant 92%↓ Non-pregnant 93%↓								12/20
Dai 2021^49^	Vaping *n* = 222 Nonuse *n* = 2849	Some days/every day					W resp 71%↓ W/o resp 68%↓	W resp 22%↓ W/o 33%↓			15/20
Goniewicz 2018^50^	Smoking *n* = 2411 Vaping *n* = 247 Nonuse *n* = 1655	Some days/every day Some days/every day Never smoked/vaped	98%↓	71%↓	91%↓	97%↓	81%↓	45%↓	25%↓	8%↓	13/20
Olivieri 2020^52^^*^	Smoking *n* = 62 Vaping *n* = 132	Daily Some days/every day	Tank 61%↓ Cartridge 52%↓								15/20
Perez 2021^51^	Smoking *n* = 961 Vaping *n* = 109 Nonuse *n* = 787	Some days/every day Some days/every day Never smoked/vaped	98%↓				82%↓				16/20
Shahab 2017^55^	Smoking *n* = 37 Vaping *n* = 37	Daily and CO verified Weekly and CO verified	97%↓		84%↓	95%↓					16/20
Smith^54^	Smoking *n* = 127 Vaping *n* = 124 Nonuse *n* = 110	Daily and CO verified Daily and CO verified No use for 6 months	92%↓		82%↓	97%↓	72%↓		13%↓	20%↓	10/20
Xia 2021^53^	Daily smoking *n* = 3176 Daily vaping *n* = 152 Ex-smoking *n* = 559		98%↓	62%↓	91%↓	96%↓	67%↓	58%↓	24%↓	18%↓	15/20
	Non-daily smoking *n* = 2400 Non-daily vaping *n* = 106 Never-smoking = 1563		71%↓	29%↓	52%↓	63%↓	76%↓	14%↓	8%↓	15%↓	

^*^Denotes studies funded by the tobacco industry CPD = cigarettes per day, CO = carbon monoxide, M = mean, SD = standardised deviation, ↓ indicates % lower among people who vaped compared to smoked, or % lower among non-users compared to people who vape, W = with respiratory symptoms, W/o = without respiratory symptoms Unless otherwise stated, results indicate urinary levels. Unless otherwise stated, results indicate urinary levels.

#### Meta-Analysis of Cross-Sectional Studies

Of the nine cross-sectional studies, seven used two overlapping data sources, one did not define frequency of vaping at recruitment, and one did not control for creatinine (Supplementary Table S12). Therefore, three studies measuring NNAL levels among people who vaped daily and smoked daily were meta-analyzed.^47,53,54^ The pooled geometric mean NNAL level was 96% lower among people who vaped daily compared to people who smoked daily (LMD = −3.21, 95% CI: −3.88, −2.54; *p* < .001; Figure 2). There was substantial heterogeneity between studies (*I*^2^ = 90%); however, all estimates were in the same direction.

### NNN Exposure

Data could not be pooled, see Supplementary Table S12.

#### Within-Group Changes

Five RCTs, reported on NNN changes after 5,^39,40^ 6,^37^ 7,^42^ and 14 days of vaping in a research facility.^36^ All reported significant reductions in levels of NNN after switching from smoking to vaping, ranging from 61%^40^ to 93%^37^ (Supplementary Table S8). NNN levels among people who smoked and had switched to vaping in two observational longitudinal studies fell by between 82%^46^ and 83%,^47^ however this was only reported to be significant in the latter^47^ (Table 1 and Supplementary Table S9).

#### Between-Group Differences

Four RCTs reported NNN levels among people who smoke who either switched to vaping or continued to smoke,^36,37,40,42^ however two studies did not report sufficient data on people who smoked to provide comparisons.^36,37^ Compared to people who continued to smoke at follow-up, NNN levels among people who switched to vaping were 80% lower after 5 days^40^; however, this was not tested for significance; and significantly lower, by 76%, after 7 days of vaping in a research facility^42^(Supplementary Table S8).

#### Cross-Sectional Studies

Three cross-sectional studies reported urinary NNN levels among people who vaped or smoked.^34,50,53^ NNN levels were significantly lower among people who vaped, by 71%^50^ to 99%,^34^ in comparison to smoked. Xia et al. reported NNN levels to be 62% lower among people who vaped daily compared to smoked daily, and 29% lower among people who vaped non-daily compared to those who smoked non-daily; neither comparison was tested for significance^53^(Table 2 and Supplementary Table S11).

Bustamante et al.,^34^ reported that saliva NNN levels were significantly lower, by 85%, among people who vaped compared to smoked^34^ (Supplementary Table S11).

### NAB and NAT Exposure

As the same studies assessed both NAB and NAT, for conciseness we report them under the same subheading.

#### Within-Group Changes

One RCT, by Round et al.,^39^ measured changes in NAB and NAT levels after switching from smoking at least 10 cigarettes per day to ad libitum vaping for 5 days in a research facility. NAB and NAT levels were significantly reduced at day 5 by 87% and 99%, respectively (Table 1, Supplementary Table S7). Two observational longitudinal studies using the PATH survey data reported that levels of NAB and NAT levels among people who smoked at wave 1 (baseline), who switched to vaping at wave 2 (12 months) fell by 89%–90%, and by 94%–96%, respectively.^45,46^ Only one study tested for significance, reporting that decreases for both NAB and NAT were significant^46^(Table 1 and Supplementary Table S9).

#### Cross-Sectional Studies

Four cross-sectional studies compared NAB and NAT levels between people who vaped or smoked.^50,53–55^ Among those who vaped compared with those who smoked, NAB and NAT levels were significantly lower, by 52%,^53^ –91%,^50^ and 63%,^53^ – 97%,^50^ respectively (Table 2 and Supplementary Table S11).

#### Meta-Analysis of Cross-Sectional Studies

The four studies that reported levels of NAB and NAT, used data from two overlapping sources (Supplementary Table S12). Therefore, two studies, each measuring NAB and NAT levels among people who smoked or vaped daily were pooled.^53,54^ Across the two studies, the pooled geometric mean NAB level was 87% lower among people who vaped daily compared to smoked daily (LMD = −2.07, 95% CI −2.81, −1.34; *p* < .001; Supplementary Figure 1). There was substantial heterogeneity between studies (*I*^2^ = 95%); however, all estimates were in the same direction. The pooled geometric mean NAT level was 94% lower among people who vaped daily compared to those who smoked daily (LMD = −2.79, 95% CI: −3.86, −1.72; *p* < .001; Supplementary figure 2). There was substantial heterogeneity between studies, although they were in the same direction (*I*^2^ = 98%).

## Vaping Versus nonuse

### NNAL Exposure

#### Between-Group Differences

Three RCTs compared NNAL levels between people who smoked and switched to vaping and people who stopped smoking without using any nicotine or tobacco products and reported no significant difference in levels between vaping and not vaping after six to seven days^37,40,42^ (Supplementary Table S8).

In observational longitudinal studies, there was a decrease in NNAL among those who quit smoking by vaping (92%–93%) and among those who quit smoking without vaping, (84%–85%).^46^ Those who switched to vaping had smoked on average 11 CPD (and had higher NNAL levels at wave 1 baseline) than those who switched to nonuse, who smoked on average 5 CPD, potentially explaining the higher percentage reduction seen among those who vaped compared to non-users at wave 2.^46^ Moreover, the mean NNAL level was still higher among those who quit smoking with vaping than those who quit without; however, this was not tested for significance. Among those who exclusively vaped at wave 1 and continued to exclusively vape at wave 2, levels of NNAL decreased by 29% but this decrease was not significant.^45^ Among those who vaped at wave 1 and quit vaping at wave 2, levels of NNAL decreased by an average 35% but this decrease was also not significant^45^ (Supplementary Table S7).

#### Cross-Sectional Studies

Six studies compared NNAL levels between people who vaped or neither vaped nor smoked.^34,49–51,53,54^ Four studies reported levels in people who neither vaped nor smoked to be 67%^53^ to 82%^51^ significantly lower compared to those who vaped (Supplementary Table S10). Bustamante et al. reported that levels of NNAL were 99% lower among people who had quit smoking without vaping for at least 6 months compared to those vaping daily; however, this was not adjusted for creatinine and was not tested for significance.^34^ Dai et al. found that among those with self-reported respiratory symptoms who neither vaped nor smoked, had 71% lower NNAL levels than those who vaped. Among those without self-reported respiratory symptoms, those who neither vaped nor smoked had 68% lower levels compared to those who vaped; these differences were not tested for significance^49^(Table 2 and Supplementary Table S10).

##### Meta-Analysis of Cross-Sectional Studies

Of the six studies that reported levels of NNAL, four used data from two overlapping sources (Supplementary Table S12). Therefore, two studies were pooled to assess NNAL between people who vaped daily and people who neither vaped nor smoked.^53,54^ Across the two studies, the geometric mean NNAL level was 80% lower among people who neither vaped nor smoked than those who vaped daily (LMD = −1.60, 95% CI: −2.27 to −0.93, *p* < .001; Figure 2). There was substantial heterogeneity between studies (*I*^2^ = 90%).

### NNN Exposure

#### Between-Group Differences

Three RCTs compared levels of NNN among people who smoked and were randomized to vaping or no nicotine or tobacco product use for 6 and 7 days in a research facility.^37,40,42^ Two reported no significant difference, and one did not test, NNN levels for significance between people who vaped and nonuse groups (Supplementary Table S8).

Two observational longitudinal studies investigated levels of NNN among people who vaped and neither vaped or smoked using wave 1 (baseline) and wave 2 (12 months) of the PATH survey.^45,46^ The decrease in NNN seen among those who quit smoking by vaping (82-83%) was greater than that seen among those who quit smoking without vaping (44%). However, mean levels of NNN were similar among people who vaped and people who did not vape after quitting smoking, although this was not tested for significance. Those who switched to vaping had smoked more CPD at wave 1, potentially explaining the higher percentage reduction seen among people who vaped compared to nonuse at wave 2.^46^ Among people who vaped at wave 1 who continued to vape at wave 2, levels of NNN increased by 5%, which was nonsignificant.^45^ Among people who vaped at wave 1 and quit vaping at wave 2, levels of NNN decreased by 23%, this was also not significant^45^ (Table 2 and Supplementary Table S11).

#### Cross-Sectional Studies

Four studies reported comparisons of urinary NNN levels between people who vaped and neither vaped nor smoked.^34,49,50,53^ Goniewicz et al. reported significant differences between the groups, with non-users having 45% lower levels compared to people who vaped.^50^ Because of variations in study designs, a meta-analysis was not feasible (Table 2 and Supplementary Table S12).

Bustamante et al. ^34^, reported that salivary NNN levels were 98% lower among people who neither vaped nor smoked compared to vaped. Differences were not tested for significance^34^( Supplementary Table S11).

### NAB and NAT Exposure

#### Between-Group Differences

Two observational longitudinal studies investigated levels of NAB and NAT among people who vaped and neither smoked or vaped- using wave 1 (baseline) and wave 2 (12 months) of the PATH survey.^45,46^ Decreases in NAB and NAT seen among those who quit smoking by vaping (NAB 89%–90%, NAT 94%–96%) were greater than those seen among those who quit smoking without vaping (NAB 63%–65%, NAT 74%–75%), likely due to the lower CPD at baseline among those who quit without vaping compared to those who quit with vaping. Among people who vaped at wave 1 and continued to vape at wave 2, levels of NAB decreased by around 7% and NAT by 5%.^45^ Among those who vaped at wave 1 and quit vaping at wave 2, levels of NAB decreased by 18% and NAT by 16%.^45^ Neither of these comparisons were tested for significance (Table 2 and Supplementary Table S9).

### Cross-Sectional

Three cross-sectional studies compared NAB and NAT levels between people who vaped and neither vaped nor smoked.^50,53,54^ Goniewicz et al. reported levels to be significantly lower among people who neither vaped nor smoked compared to people who vape by 25% for NAB and 8% for NAT.^50^ Smith et al. reported levels to be 13% for NAB, and 20% for NAT, lower among those who neither vaped nor smoked compared to vaped; however, this was not significant^54^(Table 2 and Table S11).

#### Meta-Analysis of Cross-Sectional Studies

Of the three studies, ones two used overlapping data sources (Supplementary Table S12). Therefore, data from two studies, both measuring levels among people who vaped daily and neither vaped nor smoked^53,54^ were pooled to assess NAB and NAT levels. Across the two studies, the pooled geometric mean NAB level was 26% lower among people who neither vaped nor smoked than people who vaped daily (LMD = −0.30, 95% CI: −0.59 to −0.02; *p* = .04; Supplementary Figure 3). There was substantial heterogeneity between studies (I^2^ = 68%). The pooled geometric mean NAT level was 27% lower among people who neither vaped nor smoked than people who vaped daily (LMD = −0.32, 95% CI: −0.61 to −0.03; *p* = .03; Supplementary Figure S4). There was substantial heterogeneity between studies (*I*^2^ = 69%).

## Discussion

Among 22 included studies, we found that levels of exposure to tobacco-specific nitrosamines NNAL (metabolite of NNK), NNN, NAB, and NAT were significantly reduced among people who switched from smoking to exclusive vaping in longitudinal studies (RCTs, interventional, and observational longitudinal), and were significantly lower among people who currently vaped compared to people who currently smoked in cross-sectional studies. Levels of TSNAs were also found to be similar among people who switched from smoking to vaping compared to people who switched from smoking to no use of nicotine products in longitudinal studies. Levels of TSNAs were higher among people who vaped compared to people who neither vaped nor smoked in cross-sectional studies.

Substantial reductions in NNAL levels were seen among people who switched from smoking to vaping in all longitudinal studies, and meta-analyses of three RCTs also found substantial reductions. However, there was significant heterogeneity between studies. Reductions in NNAL ranged from 55% to 84% depending on the length of time since switching from smoking to vaping. Greater reductions were seen after 24 weeks in comparison to 6 or 8 weeks after switching from smoking to exclusive vaping; this is possibly due to NNAL exposure from previous smoking that had not been fully eliminated from the body in the short-term studies. Longitudinal observational studies also reported notable decreases in NNAL among people who continued to exclusively vape for a year.^45^ This suggests continual bodily clearance of NNAL from past tobacco consumption and may suggest a longer time frame for body clearance of NNAL than previously predicted.^12,50^ There were also marked differences between study findings depending on methodology. For example, greater reductions were found in studies which were conducted in research facilities, which controlled participant’s access to smoking and vaping products,^40,42^ compared to studies in naturalistic settings^38^ with less control over participants’ smoking and vaping behaviors or potential secondhand exposures.

Longitudinal research reported significant reduction in levels of NNAL, NNN, NAB, and NAT, after people who smoked switched to vaping. However, there was variation in the magnitude of reduction between TSNA studied. For NNN, levels were reduced by between 61% and 93%, for NAB and NAT between 89% and 99%, and for NNAL 53%–84%. Differences are likely due to much shorter half-lives (30 minutes–9 hours) for NNN, NAB, and NAT. Cross-sectional research also reported substantially lower levels, often of over 90%, of NNAL, NNN, NAB, and NAT among people who vaped compared to people who smoked.

When comparing people who switched from smoking to vaping to those who switched from smoking to no use of nicotine products, one RCT reported substantially higher NNN levels among people who quit smoking by vaping compared to people who quit smoking without vaping after 5 days of switching,^40^ however no significant differences in NNN were reported after 6, 7, and 14 days in other RCTs,^36,37,42^ or after one year in observational studies.^45,46^ Reductions in NNAL, NAB, and NAT were similar or greater for people who quit smoking with vaping compared to those who quit without vaping across all studies.

Findings from cross-sectional studies differed from longitudinal studies, with NNAL, NNN, NAB, and NAT often being lower among people who neither vaped nor smoked compared to those who vaped; however, the magnitude of these differences were substantially smaller than when comparing people who vape and smoke. These differences were greatest for NNAL, which as discussed above is particularly sensitive to prior tobacco exposure. Meta-analyses of cross-sectional studies also reported significant heterogeneity, likely due to variations in methodology. For example, few cross-sectional studies included criteria on the duration of abstinence from tobacco among individuals classified as “vapers” and “non-users.” Moreover, some studies^47,54,55^ required carbon monoxide bio-verification for smoking, vaping, and nonuse status, whereas others did not. Therefore, differences between studies could be due to noncompliance. Moreover, given the differences in longitudinal and cross-sectional findings, it is possible that levels were influenced by prior tobacco exposure that had not been fully eliminated from the body. Levels may also be a result of secondhand exposure. There is some evidence that people who vape are reportedly more likely to live with someone who smokes,^56^ and could be more likely to be around people who are smoking while using outdoor smoking and vaping shelters, therefore are possibly exposed to higher levels of secondhand smoke compared to people who do not vape.

Based on NNN’s short half-life, we would expect levels to be more similar among people who vape and people who neither vaped nor smoked than those who were reported. It is possible that there was some exposure from e-liquids that contained impurities.^16,57^ However, Bustamante and colleagues^34^ suggest that saliva samples may be more sensitive to detecting NNN than urinary samples, and that differences in levels of NNN in urine and saliva samples are due to endogenous formation in the oral cavity. Previous research has also suggested endogenous synthesis of NNN among people using nicotine patches.^58,59^ Therefore, it is possible that there is some exposure from conversion of nicotine, nor nicotine, and other tobacco alkaloids into TSNAs endogenously. Moreover, it has been suggested that NNN may be affected by artefactual formation in samples from processing methods,^60^ and diet,^61^ and that plasma may be more accurate for evaluation of NNN.^62^ Therefore, there may be many confounding influences on levels of NNN that studies report.

Our findings are similar to those previously reported in other reviews.^5,22,23^ The updated search added new data on longer-term vaping, which provides new insight into the reduction of TSNA levels over time, advancing the original findings of the OHID-commissioned review.^23^ It is likely that the reduced exposure to TSNAs from vaping compared with smoking will reduce the risk of future health problems in those who switch completely from smoking to vaping. The health effects of TSNA exposure, however, cannot be viewed in isolation from other toxicants, and exposures to a range of toxicants should also be considered when assessing relative and absolute health risks of vaping. Our findings also suggest that levels of TSNAs are higher among people who vape compared to those who neither vape nor smoke. This, therefore, supports the message that people who have never smoked should not start vaping (or smoking).

More research is needed to address the limitations of the current literature. Longitudinal research would benefit from including longer follow-up periods, allowing the assessment of changes in NNAL exposure among people who vape long-term. Bio-verification of tobacco abstinence is also important for future longitudinal and cross-sectional research methodologies. When bio-verification was used, there was a range of CO levels used to determine smoking, thus the establishment of guidelines for appropriate expired CO expired breath thresholds to capture smoking, such as 3 ppm.^63^ CO measurements would be beneficial and easily incorporated into research with the technological advances and wide availability of at-home CO breathalyzers. It has also been proposed that research can bio-verify urine using acrylonitrile metabolite 2CyEMA, and when TSNAs are not the focus, NNAL, to identify combustible tobacco use.^64^ These methods may be more appropriate to capture occasional smoking, as the short half-life of CO means that it can only detect recent smoking. Cross-sectional research requires robust definitions of vaping, smoking, and nonuse and to consider the half-lives of biomarkers in these definitions. As most research was conducted in the United States; because of differing regulations of vaping products between the United States and other countries (such as the United Kingdom and the European Union, where nicotine limits are 20 mg/mL), more research from other countries is needed. Research is also needed on other toxicant exposures, specifically exposures from any vaping-specific toxicants such as glycidol.^64^ Finally, the majority of RCTs were funded by the tobacco industry, more independent research into TSNA exposure is needed.

A limitation of the present review is that it explored exclusive vaping and did not allow for the comparison of “dual users” with people who exclusively smoke or exclusively vape. Dual use can be a transitional stage between exclusive smoking and exclusive vaping, and recent estimates suggest 15%–20% of people who vape concurrently smoke cigarettes.^25^ A previous review found mixed evidence between people who exclusively vape and people who dual use.^21^ However, the definition of dual-use encompasses a wide variation in vaping patterns, therefore strict definitions of dual use, such as those outlined by Borland et al.,^24^ which are not currently used in the literature, are needed to deem any findings meaningful.^21^ Moreover, mean estimates used in meta-analyses did not control for participants' smoking and vaping characteristics, such as CPD or type of e-cigarette, which may affect levels of TSNA exposure; nor were participant demographics, such as age, sex, and ethnicity controlled for, which may affect metabolism and excretion of TSNAs. A key strength of this review is the use of meta-analysis to provide estimates of relative and absolute risk to TSNA exposure from vaping. The meta-analyses also had strict inclusion and exclusion criteria, which reduced the effects of confounders on findings.

## Conclusion

The current evidence suggests that NNAL levels are significatly lower among people who exclusively vaped compared to those who exclusively smoked, with similar differences for NAB, NAT, and NNN. Levels of TSNAs are in general higher among people who vape compared to people who neither smoke nor vape. Future high-quality research, using bio-verification and accounting for half-lives, is needed to fully assess exposure to TSNAs among people who vape. Longitudinal research is also needed to assess if TSNAs levels among people who vape can fall to levels similar to people who neither smoke nor vape in the long-term. Overall, current findings on TSNAs support the use of vapes instead of smoking for people who smoke, but also that vaping (or smoking) should not be taken up by people who have never smoked.

## Supplementary Material

A Contributorship Form detailing each author’s specific involvement with this content, as well as any supplementary data, are available online at https://academic.oup.com/ntr.

ntad156_suppl_Supplementary_Figures

ntad156_suppl_Supplementary_Tables

## References

[1] Reitsma MB, Kendrick PJ, Abdoli A, et al. Spatial, temporal, and demographic patterns in prevalence of smoking tobacco use and attributable disease burden in 204 countries and territories, 1990–2019: a systematic analysis from the Global Burden of Disease Study 2019. Lancet. 2021;397(10292):2337–2360. 34051883 10.1016/S0140-6736(21)01169-7 PMC8223261

[2] NHS. Statistics on Smoking, England 2020 - NHS Digital. Statistics on Smokings. 2020. Accessed March 1, 2022 [cited 2022 Mar 1]. https://digital.nhs.uk/data-and-information/publications/statistical/statistics-on-smoking/statistics-on-smoking-england-2020

[3] Brown KF, Rumgay H, Cox A, et al. The fraction of cancer attributable to modifiable risk factors in England, Wales, Scotland, Northern Ireland, and the United Kingdom in 2015. BJC. 2018;118(8):1130–1141. 29567982 10.1038/s41416-018-0029-6 PMC5931106

[4] International Agency for Research on Cancer. List of Classifications – IARC Monographs on the Identification of Carcinogenic Hazards to Humans. 2021. Accessed May 28, 2021. https://monographs.iarc.who.int/list-of-classifications

[5] NASEM. Public health consequences of e-cigarettes conclusions by outcome constituents of e-cigarettes. 2018. Accessed August 20, 2019. https://pubmed.ncbi.nlm.nih.gov/29894118/

[6] Hecht SS, Stepanov I, Carmella SG. Exposure and metabolic activation biomarkers of carcinogenic tobacco-specific nitrosamines. Acc Chem Res. 2016;49(1):106–114. 26678241 10.1021/acs.accounts.5b00472 PMC5154679

[7] Hecht SS. Biochemistry, biology, and carcinogenicity of tobacco-specific N- nitrosamines. Chem Res Toxicol. 1998;11(6):559–603. 9625726 10.1021/tx980005y

[8] Yuan JM, Butler LM, Stepanov I, Hecht SS. Urinary tobacco smoke constituent biomarkers for assessing risk of lung cancer. Cancer Res. 2014;74(2):401–411. 24408916 10.1158/0008-5472.CAN-13-3178 PMC4066207

[9] Edwards SH, Rossiter LM, Ding YS, et al. Tobacco-specific nitrosamines in the tobacco and mainstream smoke of U.S. commercial cigarettes. Chem Res Toxicol. 2017;30(2):540. 28001416 10.1021/acs.chemrestox.6b00268 PMC5318265

[10] Gutierrez-Torres DS, Wang L, Shiels MS, et al. Concentrations of cotinine and 4-(methylnitrosamino)-1-(3-pyridyl)-1-butanol (NNAL) in U.S. non-daily cigarette smokers. Cancer Epidemiol Biomarkers Prev. 2021;30(6):1165–1174. 33737303 10.1158/1055-9965.EPI-20-1601 PMC8172473

[11] Goniewicz ML, Havel CM, Yu L, et al. Elimination kinetics of the tobacco-specific biomarker and lung carcinogen 4-(methylnitrosamino)-1-(3-pyridyl)-1-butanol. Cancer Epidemiol Biomarkers Prev. 2009;18(12):3421–3425. 19959691 10.1158/1055-9965.EPI-09-0874 PMC2804843

[12] Hecht S, Carmella S, Murphy S, et al. Quantitation of urinary metabolites of a tobacco-specific lung carcinogen after smoking. Cancer Res. 1999;59(3):590–596. 9973205

[13] You HS, Lee J, Hwang JY, et al. Association between second-hand smoke exposure and urinary NNAL level in Korean adolescents. J Korean Med Sci. 2021;36(13):1–9. 10.3346/jkms.2021.36.e82 PMC8021977 33821591

[14] McNeill A, Brose LS, Calder R, Bauld L, Robson D. Evidence review of e-cigarettes and heated tobacco products 2018 A report commissioned by Public Health England. 2018 Accessed May 28, 2019. https://assets.publishing.service.gov.uk/government/uploads/system/uploads/attachment_data/file/684963/Evidence_review_of_e-cigarettes_and_heated_tobacco_products_2018.pdf

[15] Hartmann-Boyce J, Lindson N, Begh R, et al. Electronic cigarettes for smoking cessation. Cochrane Database Syst Rev. 2022;172022(11). 10.1002/14651858.CD010216.pub7 PMC9668543 36384212

[16] Jin XC, Wagner KA, Gardner WP, et al. Influence of nitrite on formation of tobacco-specific nitrosamines in electronic cigarette liquids and aerosols. Chem Res Toxicol. 2022 16;35(5):782–791. 35417138 10.1021/acs.chemrestox.1c00417 PMC9115799

[17] Ward AM, Yaman R, Ebbert JO. Electronic nicotine delivery system design and aerosol toxicants: a systematic review. PLoS One. 2020 1;15(6):e0234189. 32497139 10.1371/journal.pone.0234189 PMC7272070

[18] Goniewicz ML, Knysak J, Gawron M, et al. Levels of selected carcinogens and toxicants in vapour from electronic cigarettes. Tob Control. 2014;23:133–139. 23467656 10.1136/tobaccocontrol-2012-050859 PMC4154473

[19] Committee on Toxicity of Chemicals in Food Consumer Products and The Environment. TOX/2018/1 Potential toxicological risks from electronic nicotine (or non-nicotine) delivery systems (e-cigarettes). Preparation for further discussion papers Background. 2018 Accessed March 10, 2022. https://cot.food.gov.uk/sites/default/files/2020-09/COT%20E%28N%29NDS%20statement%202020-04.pdf

[20] Jacobson K, Martinez J, Larroque S, Jones IW, Paschke T. Nicotine pharmacokinetics of electronic cigarettes: a pooled data analysis from the literature. Toxicol Rep. 2021 1;8:84–95. 10.1016/j.toxrep.2020.12.016 PMC7786013 33437651

[21] Hartmann-Boyce J, Butler AR, Bullen C, et al. Biomarkers of potential harm in people switching from smoking tobacco to exclusive e-cigarette use, dual use or abstinence: secondary analysis of Cochrane systematic review of trials of e-cigarettes for smoking cessation. Addiction. 2023;118(3):539–545. 36208090 10.1111/add.16063 PMC10092879

[22] Akiyama Y, Sherwood N. Systematic review of biomarker findings from clinical studies of electronic cigarettes and heated tobacco products. Toxicol Rep. 2021;8:282–294. 33552927 10.1016/j.toxrep.2021.01.014 PMC7850959

[23] McNeill A, Simonavičius E, Robson D, et al. Nicotine Vaping in England: 2022 Evidence Update. 2022 Accessed September 30, 2022. https://www.gov.uk/government/publications/nicotine-vaping-in-england-2022-evidence-update

[24] Borland R, Murray K, McNeill A, et al. A new classification system for describing concurrent use of nicotine vaping products alongside cigarettes (so‐called “dual use”): findings from the ITC‐4 Country Smoking and Vaping wave 1 Survey. Addiction. 2019;114(suppl 1):24–34. 10.1111/add.14570 PMC6669110 30702175

[25] McNeill A, Brose L, Calder R, Simonavicius E, Robson D. Vaping in England: an evidence update including vaping for smoking cessation, 2021 A report commissioned by Public Health England. 2021 Accessed March 10,, 2021. https://www.gov.uk/government/publications/vaping-in-england-evidence-update-february-2021.

[26] McNeill A, Brose LS, Calder R, Bauld L, Robson D. Vaping in England: an evidence update including mental health and pregnancy, 2020 Accessed July 13, 2020. https://assets.publishing.service.gov.uk/government/uploads/system/uploads/attachment_data/file/869401/Vaping_in_England_evidence_update_March_2020.pdf.

[27] Sterne JA, Hernán MA, Viswanathan M, et al. ROBINS-I: a tool for assessing risk of bias in non-randomised studies of interventions. BMJ. 2016;12:355. 10.1136/bmj.i4919 PMC5062054 27733354

[28] Wells G, O’Connell D, Peterson J, Welch V, Losos M, Tugwell P. The Newcastle-Ottawa Scale (NOS) for assessing the quality of nonrandomised studies in meta-analyses. Ottawa Hospital Research Institute. 2013. https://www.ohri.ca//programs/clinical_epidemiology/oxford.asp

[29] Wirsching J, Graßmann S, Barth E, et al. Development and reliability assessment of a new quality appraisal tool for cross-sectional studies using biomarker data (BIOCROSS). BMC Med Res Methodol. 2018;18(1):1–8. 30400827 10.1186/s12874-018-0583-x PMC6219097

[30] Higgins JPT, White IR, Anzures-Cabrera J. Meta-analysis of skewed data: combining results reported on log-transformed or raw scales. Stat Med. 2008;27(29):6072–6092. 18800342 10.1002/sim.3427 PMC2978323

[31] Deeks JJ, Higgins JPT, Altman DG, eds. Chapter 10: Analysing data and undertaking meta-analyses. In: Higgins JPT, Thomas J, Chandler J, Cumpston M, Li T, Page MJ, Welch VA, eds. Cochrane Handbook for Systematic Reviews of Interventions version 6.3 (updated February 2022). Cochrane, 2022. www.training.cochrane.org/handbook

[32] Cochrane Training. RevMan. Accessed November 7, 2022. https://training.cochrane.org/online-learning/core-software/revman

[33] Robson D, Brose L, McNeill A, et al. A systematic review of the health risks and health effects of vaping. PROSPERO. 2020. https://www.crd.york.ac.uk/prospero/display_record.php?RecordID=215915.

[34] Bustamante G, Ma B, Jensen J, et al. Presence of the carcinogen N’-nitrosonornicotine in saliva of E-cigarette users. Chem Res Toxicol. 2018;31(8):731–738. 30019582 10.1021/acs.chemrestox.8b00089 PMC8556657

[35] Edmiston JS, Webb KM, Sarkar Pharm MM, et al. Biomarkers of exposure and biomarkers of potential harm in adult smokers who switch to e-vapor products relative to cigarette smoking in a 24-week, randomized, clinical trial. Nicotine Tob Res. 2022 15;24(7):1047–1054. 35134961 10.1093/ntr/ntac029 PMC9199942

[36] Morris P, McDermott S, Stevenson M, et al. Reductions in biomarkers of exposure to selected harmful and potentially harmful constituents following exclusive and partial switching from combustible cigarettes to myblu ^TM^ electronic nicotine delivery systems (ENDS). Intern Emerg Med. 2022 1;17(2):397–410. 34435305 10.1007/s11739-021-02813-w PMC8964552

[37] Cohen G, Goldenson NI, Bailey PC, Chan S, Shiffman S. Changes in biomarkers of cigarette smoke exposure after 6 days of switching exclusively or partially to use of the JUUL system with two nicotine concentrations: a randomized controlled confinement study in adult smokers. Nicotine Tob Res. 2021 5;23(12):2153–2161. 34161586 10.1093/ntr/ntab134 PMC8570669

[38] Hatsukami D, Meier E, Norton K, et al. A randomized clinical trial examining the effects of instructions for electronic cigarette use on smoking-related behaviors, and biomarkers of exposure. Nicotine Tob Res. 2019 24;22(9):1524–1532. 10.1093/ntr/ntz233 PMC7443587 31828315

[39] Round EK, Chen P, Taylor AK, Schmidt E. Biomarkers of tobacco exposure decrease after smokers switch to an E-cigarette or nicotine gum. Nicotine Tob Res. 2019;21(9):1239–1247. 30202883 10.1093/ntr/nty140 PMC6698949

[40] Jay J, Pfaunmiller EL, Huang NJ, Cohen G, Graff DW. Five-day changes in biomarkers of exposure among adult smokers after completely switching from combustible cigarettes to a nicotine-salt pod system. Nicotine Tob Res. 2020;22(8):1285–1293. 31688930 10.1093/ntr/ntz206 PMC7364828

[41] Pulvers K, Nollen NL, Benowitz NL, et al. Effect of pod e-cigarettes vs cigarettes on carcinogen exposure among African American and Latinx smokers: a randomized clinical trial. JAMA Netw Open. 2020;3(11):e2026324. 33206193 10.1001/jamanetworkopen.2020.26324 PMC7675102

[42] McEwan M, Gale N, Proctor CJ, et al. A randomized controlled study in healthy participants to explore the exposure continuum when smokers switch to a tobacco heating product or an E-cigarette relative to cessation. Toxicol Rep. 2021;8:994–1001. 34026564 10.1016/j.toxrep.2021.05.003 PMC8131274

[43] Goniewicz ML, Gawron M, Smith DM, et al. Exposure to nicotine and selected toxicants in cigarette smokers who switched to electronic cigarettes: a longitudinal within-subjects observational study. Nicotine Tob Res. 2017;19(2):160–167. 27613896 10.1093/ntr/ntw160 PMC5234360

[44] Pulvers K, Emami AS, Benowitz NL, et al. Tobacco consumption and toxicant exposure of cigarette smokers using electronic cigarettes. Nicotine Tob Res. 2018;20(2):206–214. 28003511 10.1093/ntr/ntw333 PMC6251645

[45] Dai H, Benowitz NL, Achutan C, et al. Exposure to toxicants associated with use and transitions between cigarettes, e-cigarettes, and no tobacco. JAMA Netw Open. 2022 1;5(2):e2147891. 35142830 10.1001/jamanetworkopen.2021.47891 PMC8832174

[46] Anic GM, Rostron BL, Christensen CH, et al. Changes in biomarkers of tobacco exposure among cigarette smokers transitioning to ENDS use: the Population Assessment of Tobacco and Health Study, 2013-2015. Int J Environ Res Public Health. 2022;19(3):1462. 35162490 10.3390/ijerph19031462 PMC8835100

[47] Carroll DM, Wagener TL, Stephens LD, et al. Biomarkers of exposure in ENDS users, smokers, and dual users of American Indian Descent. Tob Regul Sci. 2018;4(2):3–15. 10.18001/TRS.4.2.1 PMC6792294 32205902

[48] Coleman SRM, Bunn JY, Tyndale RF, et al. Use of electronic nicotine delivery systems (ENDS) among U.S. women of reproductive age: prevalence, reported reasons for use, and toxin exposure. Prev Med. 2021;152(Pt 2):106582. 33930436 10.1016/j.ypmed.2021.106582 PMC8545704

[49] Dai H, Khan AS. A longitudinal study of exposure to tobacco-related toxicants and subsequent respiratory symptoms among U.S. adults with varying E-cigarette use status. Nicotine Tob Res. 2020;22(suppl 1):S61–S69. 33320254 10.1093/ntr/ntaa180 PMC8628872

[50] Goniewicz ML, Smith DM, Feng J, et al. Comparison of nicotine and toxicant exposure in users of electronic cigarettes and combustible cigarettes. JAMA Netw Open. 2018;1(8):e185937–e185937. 30646298 10.1001/jamanetworkopen.2018.5937 PMC6324349

[51] Perez MF, Mead EL, Atuegwu NC, et al. Biomarkers of toxicant exposure and inflammation among women of reproductive age who use electronic or conventional cigarettes. J Womens Health. 2021;30(4):539–550. 10.1089/jwh.2019.8075 PMC8064962 33534627

[52] Oliveri D, Liang Q, Sarkar M. Real-world evidence of differences in biomarkers of exposure to select harmful and potentially harmful constituents and biomarkers of potential harm between adult e-vapor users and adult cigarette smokers. Nicotine Tob Res. 2020;22(7):1114–1122. 31563966 10.1093/ntr/ntz185 PMC7291803

[53] Xia B, Blount BC, Van Bemmel DM, et al. Tobacco-specific nitrosamines (NNAL, NNN, NAT, and NAB) exposures in the US population assessment of tobacco and health (PATH) study wave 1 (2013-2014). Nicotine Tob Res. 2021;23(3):573–583. 32716026 10.1093/ntr/ntaa110 PMC7885786

[54] Smith DM, Shahab L, Sobczak A, et al. Differences in exposure to nicotine, tobacco-specific nitrosamines, and volatile organic compounds among electronic cigarette users, tobacco smokers, and dual users from three countries. Toxics. 2020;8(4):14. 33066428 10.3390/toxics8040088 PMC7712026

[55] Shahab L, Goniewicz ML, Alwis KU, et al. Nicotine, carcinogen, and toxin exposure in long-term E-cigarette and nicotine replacement therapy users: a cross-sectional study. Ann Intern Med. 2017;166(6):390–400. 28166548 10.7326/M16-1107 PMC5362067

[56] Li D, Shi H, Xie Z, Bansal-Travers M, et al. Home smoking and vaping policies among US adults: results from the population assessment of tobacco and health (PATH) study, wave 3. Prev Med. 2020;139:106215. 32693178 10.1016/j.ypmed.2020.106215 PMC7494576

[57] Flora JW, Wilkinson CT, Sink KM, McKinney DL, Miller JH. Nicotine-related impurities in e-cigarette cartridges and refill e-liquids. J Liq Chromatogr Relat Technol. 2017;39(17–18):821–829.

[58] Stepanov I, Carmella SG, Lerman C, et al. Evidence for endogenous formation of Nʹ-nitrosonornicotine in some long-term nicotine patch users. Nicotine Tob Res. 2009;11(1):99. 19246447 10.1093/ntr/ntn004 PMC2734288

[59] Knezevich A, Muzic J, Hatsukami DK, Hecht SS, Stepanov I. Nornicotine nitrosation in saliva and its relation to endogenous synthesis of nʹ-nitrosonornicotine in humans. Nicotine Tob Res. 2013;15(2):591–595. 22923602 10.1093/ntr/nts172 PMC3611998

[60] Kavvadias D, Scherer G, Cheung F, et al. Determination of tobacco-specific N-nitrosamines in urine of smokers and non-smokers Tobacco-specific N-nitrosamines in urine of smokers and non-smokers D. Biomarkers. 2009;14(8):547–553. 19747086 10.3109/13547500903242883

[61] Tyroller S, Zwickenpflug W, Richter E. New sources of dietary myosmine uptake from cereals, fruits, vegetables, and milk. J Agric Food Chem. 2002;50(17):4909–4915. 12166981 10.1021/jf020281p

[62] Pluym N, Scherer G, Edmiston JS, et al. Assessment of the exposure to NNN in the plasma of smokeless tobacco users. Chem Res Toxicol. 2022;35(4):663–669. 35298127 10.1021/acs.chemrestox.1c00431 PMC9019808

[63] Cropsey KL, Trent LR, Clark CB, et al. How low should you go? Determining the optimal cutoff for exhaled carbon monoxide to confirm smoking abstinence when using cotinine as reference. Nicotine Tob Res. 2014;16(10):1348–1355. 24891552 10.1093/ntr/ntu085 PMC4207872

[64] Goniewicz ML. Biomarkers of electronic nicotine delivery systems (ENDS) use. Addict Neurosci. 2023;6:100077. 37089248 10.1016/j.addicn.2023.100077 PMC10121191

