# Supplementary material for: Exposure to Tobacco-Specific Nitrosamines Among People Who Vape, Smoke, or do Neither: A Systematic Review and Meta-Analysis
Source: Nicotine Tob Res. 2023 Aug 24;26(3):257–69. doi: 10.1093/ntr/ntad156 (PMC10882431; doi:10.1093/ntr/ntad156)
Supplement: ntad156_suppl_Supplementary_Figures [file ntad156_suppl_supplementary_figures.docx]

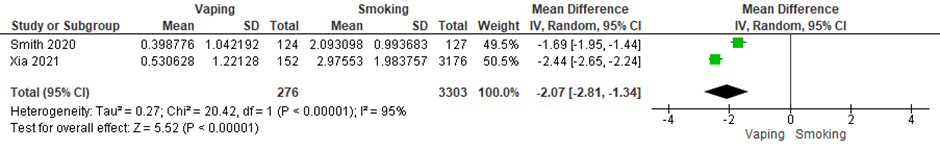
**Supplementary figure 1: Meta-analysis of cross-sectional studies reporting on urinary NAB levels between vapers and smokers.**


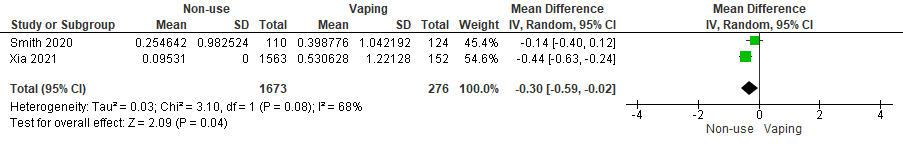
**Supplementary figure 3: Meta-analysis of cross-sectional studies reporting on urinary NAB levels between vapers and non-users.**


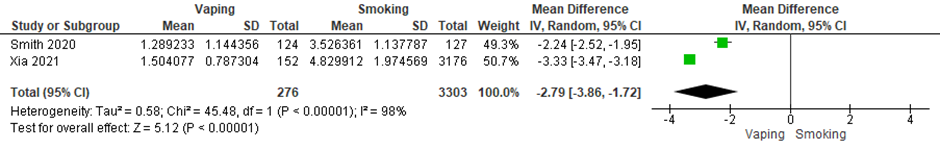
**Supplementary figure 2: Meta-analysis of cross-sectional studies reporting on urinary NAT levels between vapers and smokers.**


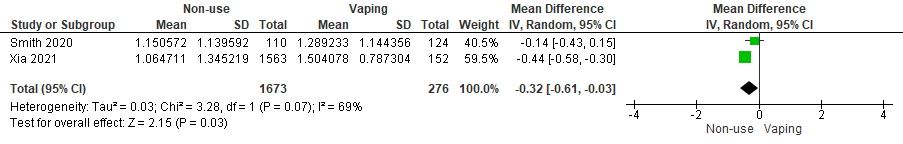
**Supplementary figure 4: Meta-analysis of cross-sectional studies reporting on urinary NAT levels between vapers and non-users.**
